# Microorganism-Based Biological Products for Agriculture: From Strain Selection to Production Organization

**DOI:** 10.3390/microorganisms14040775

**Published:** 2026-03-29

**Authors:** Amankeldi K. Sadanov, Gul Baimakhanova, Baiken B. Baimakhanova, Saltanat Orazymbet, Irina A. Ratnikova, Irina Smirnova, Gulzat S. Aitkaliyeva, Ayaz M. Belkozhayev, Bekzhan D. Kossalbayev

**Affiliations:** 1 LLP “Research and Production Center for Microbiology and Virology”, Almaty 050010, Kazakhstan; a.sadanov1951@gmail.com (A.K.S.); bgulb@mail.ru (G.B.); bbbayken@mail.ru (B.B.B.); saltanatmikro@gmail.com (S.O.); iratnikova@list.ru (I.A.R.); iesmirnova@mail.ru (I.S.); 2 Department of Chemical and Biochemical Engineering, Geology and Oil-Gas Business Institute Named After K. Turyssov, Satbayev University, Almaty 050043, Kazakhstan; a.belkozhayev@satbayev.university; 3 M.A. Aitkhozhin Institute of Molecular Biology and Biochemistry, Almaty 050012, Kazakhstan; 4 Ecology Research Institute, Khoja Akhmet Yassawi International Kazakh Turkish University, Turkistan 161200, Kazakhstan; 5 Faculty of Biology and Biotechnology, Al-Farabi Kazakh National University, Al-Farabi Ave. 71, Almaty 050038, Kazakhstan

**Keywords:** biofertilizers, biopesticides, PGPM, PGPR, microbial consortia, fermentation, formulation, quality control, sustainable agriculture

## Abstract

Plant growth-promoting microorganisms (PGPMs) and microbial biocontrol agents have emerged as key tools for improving crop productivity while maintaining environmental sustainability. However, central questions remain regarding which factors determine their consistent field performance and how these factors interact under real agronomic conditions. Previous research has demonstrated that PGPMs enhance nutrient acquisition, regulate phytohormone balance, improve stress tolerance, and suppress plant pathogens through diverse biochemical and ecological mechanisms. Advances in omics technologies, genome mining, and synthetic microbial communities have further expanded understanding of their functional potential. Nevertheless, many studies rely on laboratory-scale experiments or short-term trials, with limited multi-season and cross-regional validation. This gap contributes to inconsistent field outcomes and restricts large-scale agricultural adoption. Long-term multi-season validation and reproducibility assessment remain essential priorities for improving reliability of microbial agricultural products. This review synthesizes recent advances in PGPM-based biofertilizers and microbial biocontrol technologies, critically examining their mechanisms of action, scalability constraints, formulation challenges, and regulatory limitations. It identifies major translational barriers, including context dependency, mechanistic uncertainties, reproducibility gaps, and insufficient systems-level integration.

## 1. Introduction

Agriculture is entering a period in which productivity must increase, yet environmental pressure must decrease. For decades, crop yields have been strongly supported by mineral fertilizers and chemical pesticides. While effective, these inputs have also contributed to soil degradation, nutrient imbalances, reduced microbial diversity, and contamination of surrounding ecosystems. As a result, sustainable agricultural systems increasingly rely on biological solutions that work with natural processes rather than replacing them [1,2]. Microorganism-based biological products represent one of the most dynamic directions in this transition [3,4]. Beneficial bacteria and fungi can mobilize nutrients, stimulate plant growth, suppress pathogens, and enhance plant tolerance to environmental stress [5,6]. Unlike synthetic inputs, microbial products function through biochemical transformations and ecological interactions in the soil–plant system. Their activity contributes not only to crop productivity but also to soil health and long-term ecosystem stability [7,8]. The scientific understanding of microbial bioproducts has evolved significantly. Early studies often focused primarily on visible plant growth stimulation [9]. Today, research increasingly emphasizes measurable mechanisms such as nitrogen fixation, phosphorus solubilization, siderophore production, phytohormone synthesis, and Induced systemic resistance (ISR) [10,11,12]. In addition, the potential of certain strains to partially substitute mineral fertilizers is now evaluated using nutrient fraction analysis and agronomic equivalence approaches. This shift reflects a broader movement toward evidence-based integration of microbial inputs into modern nutrient management systems [13,14]. Microorganism-based agricultural products can be grouped into several functional categories, including biofertilizers [15], biopesticides [16], PGPMs, and multistrain microbial consortia [17,18]. Although their mechanisms differ, these products often share interconnected modes of action. For example, a strain selected for nutrient mobilization may simultaneously enhance stress tolerance or pathogen resistance [19,20]. Increasingly, multifunctional formulations are being developed to address complex agronomic challenges under variable field conditions [21]. However, identifying a beneficial microorganism is only the first step. The development of a reliable biological product requires a systematic pathway that includes strain isolation, functional screening, molecular characterization, optimization of cultivation conditions, formulation design, quality control, and regulatory assessment [22,23,24,25]. Genomic and omics technologies now allow for precise taxonomic identification and prediction of functional traits, improving strain selection and reducing biosafety risks [26]. At the same time, industrial production introduces additional challenges related to scalability, genetic stability, stress tolerance during fermentation, and shelf life preservation [27,28]. Bridging the gap between laboratory discovery and consistent field performance remains a central issue. Environmental factors such as soil type, climate, native microbiota composition, and agronomic practices can strongly influence efficacy [29]. Therefore, successful implementation depends on integrating microbiological knowledge with production engineering, formulation science, and practical agronomy. Understanding microorganism-based biological products as complete technological systems from strain selection to production organization and field validation provides a framework for developing stable, effective, and economically viable solutions [30,31]. Such an integrated perspective is essential for advancing sustainable agriculture while maintaining productivity under increasingly complex environmental conditions. This review synthesizes current knowledge on microorganism-based biological products for agriculture, examining their functional categories, sources and criteria for strain selection, molecular and genomic characterization strategies, optimization of cultivation and fermentation processes, formulation and quality control approaches, and production organization models. By connecting biological mechanisms with technological development and practical implementation, the review aims to present a coherent pathway from microbial discovery to scalable and field-validated agricultural applications, while outlining existing limitations and future research directions.

## 2. Types of Microorganism-Based Biological Products in Agriculture

### 2.1. Biofertilizers

**Figure 1:**
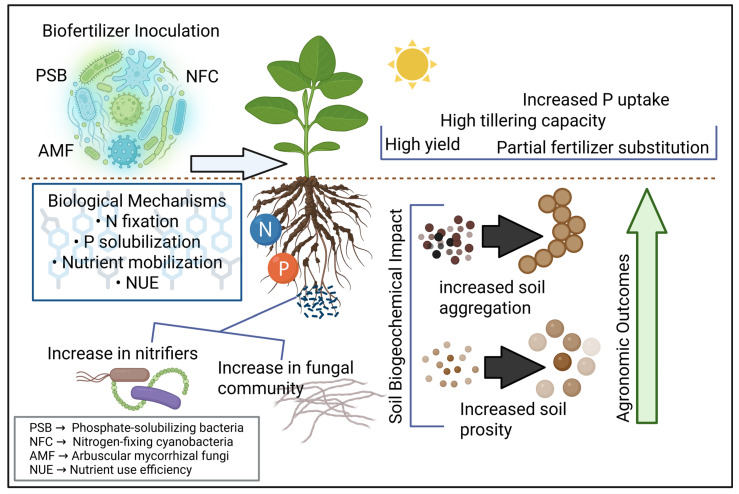
Conceptual framework of biofertilizer inoculation effects on plant performance and soil biogeochemical processes. PSB, NFC, and AMF enhance nutrient mobilization, nitrogen fixation, and phosphorus uptake, improving nutrient use efficiency and plant growth. Created with BioRender.com (License No. AA29DABWJE).

Biofertilizers are microbial-based products designed to enhance nutrient availability, uptake, and utilization efficiency in plants [32]. Biofertilizer inoculation refers to the deliberate application of beneficial microorganisms to seeds, plant roots, or soil to stimulate nutrient mobilization through biological processes. Their primary function is to supply nutrients through biological processes, such as biological nitrogen fixation, phosphorus solubilization, or mobilization of nutrients already present in soil reserves [33,34]. Unlike general plant growth stimulants, true biofertilizers focus specifically on improving plant nutrition through measurable biochemical and microbiological mechanisms [35,36]. Figure 1 illustrates the main mechanisms and functional pathways through which microbial biofertilizers enhance plant nutrition and soil nutrient availability.

Recent studies evaluate biofertilizers not only by their ability to stimulate plant growth, but also by quantifying their contribution to nutrient cycling and their potential to substitute mineral fertilizers. For example, Beltran-Medina et al. (2023) [37] demonstrated that inoculation with *Rhizobium* sp. B02, combined with 50% of the recommended phosphorus (P) fertilizer rate, increased the labile inorganic P fraction in soil by 14% and improved maize growth and grain yield. Yield increased by 696 kg ha^−1^ compared with the reduced-P control and reached levels comparable to full P fertilization without inoculation.

Nitrogen-fixing cyanobacteria (NFC) represent a complementary biofertilizer approach, capable of partially substituting synthetic nitrogen inputs while influencing nitrogen losses and soil nitrogen dynamics. In a two-year field experiment, Song et al. (2021) compared conventional urea fertilization with graded urea replacement by NFC and quantified ammonium-N, nitrate-N, total dissolved N, and dissolved organic N in percolated irrigation water to assess leaching losses and nitrogen-use efficiency. The study showed that full replacement of urea with NFC resulted in lower yield compared with conventional fertilization. However, replacing 50% of urea with NFC maintained rice yield while reducing total dissolved nitrogen leaching by approximately 37% [38]. Liang et al. (2024) showed that partial substitution of mineral nitrogen fertilizer with NFC altered microbial community composition and enhanced soil carbon utilization dynamics [39].

A two-season field evaluation of a dual-strain phosphate-solubilizing bacterial (PSB) inoculant (*Priestia megaterium* and *Bacillus subtilis*) tested its performance under contrasting phosphorus sources and application rates. The results showed that grain yield did not significantly differ between inoculated and non-inoculated treatments, despite clear variations in root traits and microbial responses across seasons. Multivariate analysis indicated that seasonal and climatic variation explained a larger proportion of yield variability than inoculation itself [40].

Beyond bacterial inoculants, fungal biofertilizers also play a significant role. Arbuscular mycorrhizal fungi (AMF) enhance nutrient uptake particularly phosphorus by expanding the effective root absorption zone and can additionally improve soil structure and grain nutritional quality. Akbar et al. (2023) reported that AMF inoculation in wheat increased tillering and was associated with higher soil organic carbon and available P and K, along with improvements in grain nutritional traits [41]. Biofertilizers can significantly improve nutrient cycling and plant productivity. However, their field performance strongly depends on environmental conditions, soil characteristics, and long-term validation across multiple growing seasons.

### 2.2. Biopesticides and Biocontrol Agents

Microbial biopesticides and biocontrol agents are primarily developed to reduce pest and pathogen pressure in crops [42,43]. They act either through direct mechanisms such as antibiosis, competition for nutrients and space, or parasitism or indirectly by stimulating plant defense responses, including induced systemic resistance (ISR) and defense priming [44,45]. First, it is necessary to identify strains that exhibit stable and strong antagonistic activity under relevant ecological conditions [46]. Second, microbial cells or their bioactive metabolites must be formulated in a way that ensures survival during storage and under field stresses such as ultraviolet radiation, temperature fluctuations, and desiccation [47,48]. Third, product performance must be validated under realistic agronomic conditions, considering infection pressure, application dose, root or leaf colonization capacity, and compatibility with other agricultural inputs [49,50].

At the strain level, Chen et al. (2022) describes *Bacillus velezensis* SDTB038 as a genomically supported biocontrol candidate. Whole-genome sequencing identified multiple secondary metabolite biosynthesis clusters consistent with antimicrobial activity, and in vitro assays confirmed pathogen inhibition. In pot experiments, optimized application reduced disease severity, decreased ROS accumulation, and upregulated cold-responsive genes, indicating combined biocontrol and plant growth-promoting effects [51].

Du et al. (2026) implemented a pipeline in which pathogen challenge was used to selectively enrich rhizosphere bacteria with antagonistic potential. *Bacillus amyloliquefaciens* E2 demonstrated strong in vitro inhibition of the target pathogen, effective colonization of roots and stems in pot experiments, and high disease control efficacy [52].

At the formulation stage, fungal biopesticides illustrate that product performance is closely linked to materials engineering. Recent studies on microencapsulation of *Beauveria bassiana* conidia using polymer-based systems demonstrate how formulation parameters directly influence stability and efficacy. Optimized spray-dried or ionically gelled systems improved conidia loading, controlled moisture and water activity, enhanced resistance to thermal and UV stress, and increased insecticidal performance compared with non-formulated propagules [53].

Kamouni et al. (2025) advances a different encapsulation approach ionic-gelation bead based on carboxymethylcellulose (CMC) cross-linked with Al^3+^ or Fe^3+^ and adds a deeper structural characterization toolchain. The paper reports that Al-based beads provided markedly higher conidia loading and strong protection under high UV and extreme heat, illustrating how cross-link chemistry can change microstructure and thereby protective performance. Methodologically, this study is valuable because it treats microencapsulation as an engineered system with measurable physical properties that can be optimized, rather than as a black box carrier [54].

Intana et al. (2023) addresses a complementary formulation challenge focused on operational usability and shelf life under farmer-relevant storage conditions. The study developed an emulsion-based formulation of *Trichoderma asperelloides* PSU-P1 conidia using a defined oil–emulsifier–water ratio and evaluated antifungal activity in vitro, in vivo disease suppression, and conidial viability during storage under ambient and cool conditions. The formulation maintained antifungal efficacy over several months and demonstrated improved suspension stability compatible with conventional sprayer systems. This work highlights the importance of aligning biological performance with storage stability and practical field application in biopesticide development [55]. Representative studies and methodological advances are summarized in Table 1. These studies demonstrate that successful biopesticide development requires integration of genomics, microbiome-guided strain discovery, and advanced formulation technologies to ensure reliable disease suppression under field conditions.

### 2.3. Plant Growth-Promoting Microorganisms (PGPM)

Although the terms biofertilizers and PGPM are sometimes used interchangeably, biofertilizers are generally defined as microbial products primarily aimed at improving plant nutrient availability, whereas PGPM represent a broader functional category that includes microorganisms promoting plant growth through multiple mechanisms such as phytohormone production, stress tolerance, and pathogen suppression.

**Figure 2:**
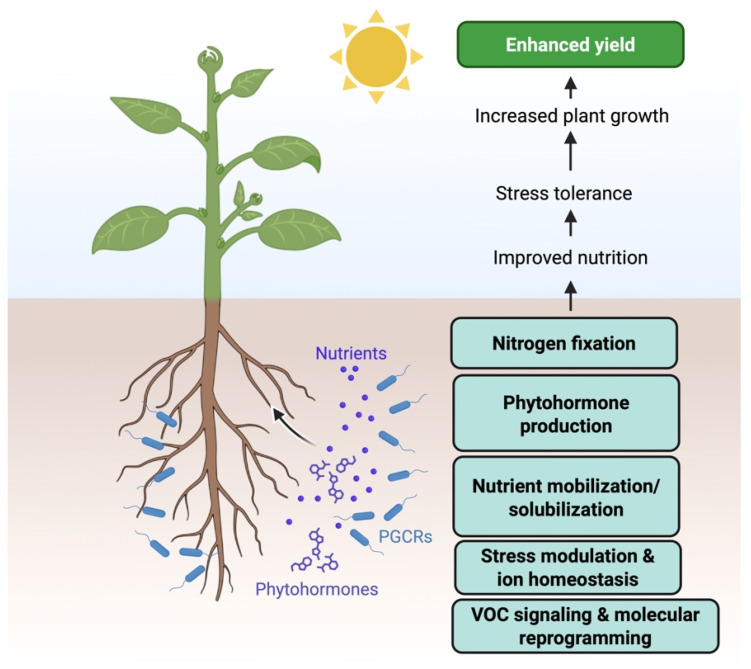
Functional framework of plant growth-promoting microorganisms. PGPMs enhance plant growth through nitrogen fixation, phytohormone production, nutrient mobilization, and stress modulation. These mechanisms improve nutrient uptake, stress tolerance, and overall plant development, ultimately leading to enhanced crop yield. Created with BioRender.com (License No. GP29E55F25).

PGPMs, including plant growth-promoting rhizobacteria (PGPR) and beneficial endophytes, are primarily characterized by their ability to improve plant growth, vigor, and tolerance to environmental stress [61,62]. They act by modifying plant physiology through microbe–plant interactions that influence signaling pathways, nutrient allocation, and metabolic balance [63]. Research in this field has identified several key functional mechanisms. These mechanisms include ethylene regulation via ACC deaminase, modulation of phytohormone pathways, VOC-mediated effects on root development and stress responses, maintenance of ion homeostasis under salinity or metal stress, and molecular pathway adjustments detectable through omics analyses [64,65] (Figure 2).

Gupta et al. (2021) screened bacterial isolates for ACC deaminase activity, identified two high-activity *Bacillus* strains, and confirmed the functional gene by PCR. Inoculation mitigated salt stress in pea, improving biomass, biochemical parameters, antioxidant enzyme activity, and expression of stress-related genes. The study demonstrates a clear validation pathway linking enzymatic screening and genetic confirmation to measurable plant physiological and molecular responses [66].

Daraz et al. (2023) isolated PGPR from salt- and cadmium-stressed soils, selected strains tolerant across wide pH/salinity/Cd ranges and tested them on *Brassica* under single and dual stress. The study reports improved biomass parameters, increased K and Ca uptake with restricted Na and Cd in shoots, and reduced Cd translocation an explicit “ion homeostasis” mechanism rather than a generic growth effect [67]. This advances PGPM product logic by linking strain selection to stress-specific physiological endpoints that are directly relevant to saline or contaminated soils.

Endophytic PGPM can act through deeper host signaling reprogramming, but they also raise higher demands for safety and precise taxonomic characterization. Hwang et al. (2025) examined an endophytic bacterium and showed that inoculated plants experienced less oxidative stress under salt and drought, with lower induction of H_2_O_2_, electrolyte leakage, MDA, and proline under stress, consistent with transcriptome findings and improved growth outcomes in both a model plant and a vegetable host. The contribution here is the mechanistic convergence of transcriptomic and biochemical stress markers, making it easier to design QC assays or biomarkers for “stress alleviation” PGPM claims [68].

Proteomic approaches are increasingly used to connect PGPM-induced plant phenotypes with underlying molecular mechanisms. In a recent study, soybean-associated *Bacillus* strains selected for growth-promoting and biocontrol traits were evaluated using shotgun proteomics. Strain-specific changes in the soybean leaf proteome were identified and linked to observable outcomes such as enhanced germination or increased leaf area. Enriched protein groups were associated with amino acid metabolism and biosynthetic pathways [69].

Volatile organic compounds (VOCs) represent a distinct PGPM mechanism because they can promote plant growth without direct root colonization. Under controlled salinity conditions, bacterial VOCs enhanced root length, lateral root formation, and fresh weight, while also modifying physiological markers such as proline levels and sodium accumulation in plant tissues. Chemical profiling using SPME–GC–MS enabled partial identification of active compounds. This integrated phenotype–physiology–chemistry framework strengthens the mechanistic basis for VOC-mediated growth promotion and supports their potential development into applied PGPM technologies [70]. PGPM research increasingly links microbial traits with measurable physiological and molecular plant responses, enabling the development of strains tailored to specific stress conditions and agricultural environments.

### 2.4. Microbial Consortia and Multistrain Products

Microbial consortia and multistrain products attempt to solve a core limitation of single-strain inoculants: agricultural environments are heterogeneous, and single organisms often cannot provide stable, multi-functional performance across variable nutrient, water, pest, and microbiome contexts. Consortia therefore aim for complementary functions and ecological robustness [71,72,73].

Cirillo et al. (2023) tested a two-member consortium combining a nitrogen-fixing bacterium, *Azotobacter chroococcum* 76A, and a beneficial fungus, *Trichoderma afroharzianum* T22, designed to provide complementary growth-promoting effects. The study assessed tomato performance under reduced water and nitrogen conditions, measuring yield, physiological parameters such as SPAD values and leaf water potential, and rhizosphere microbial diversity. While yield increases were evident under optimal inputs, co-inoculation under reduced inputs improved shoot biomass and plant physiological status and altered rhizosphere microbial composition [74]. Consortia are increasingly explored as strategies to reduce chemical fertilizer inputs while maintaining crop productivity. Open-field trials in tomato demonstrated that synthetic communities composed of free-living nitrogen-fixing bacteria and mycorrhizal–bacterial mixtures can sustain marketable yields under reduced fertilization while enhancing rhizosphere biodiversity. Metabarcoding analyses revealed shifts in resident microbial communities and highlighted methodological limits in species-level resolution [75]. At field scale, consortium performance is often stabilized through integration with non-microbial amendments such as biochar. Multi-year trials combining synthetic communities with AMF reported synergistic improvements in maize physiology and kernel metabolite profiles, without significant disruption of native soil microbial diversity [76]. Consortia have also been evaluated alongside herbicides to reduce chemical inputs while maintaining weed control and crop yield. Field experiments showed that selected synthetic communities, when combined with reduced herbicide doses, suppressed weeds and mitigated negative effects on wheat performance [77,78]. Microbial consortia represent a promising strategy for improving stability and multifunctionality of biological products, but rational design and ecological compatibility remain key challenges.

## 3. Sources and Selection of Microbial Strains

### 3.1. Natural Sources of Agricultural Microorganisms

Natural sources of agricultural microorganisms can be viewed as ecological niches that shape microbial traits through specific environmental pressures [79]. These environments influence properties such as colonization capacity, stress tolerance, and interactions with plants and other microbes [80]. Microorganisms isolated from the rhizosphere are typically adapted to root colonization and nutrient mobilization [81,82]. Endophytes are selected for their ability to survive within plant tissues, often under host immune regulation. Phyllosphere microbes must tolerate ultraviolet radiation, desiccation, and competition on leaf surfaces [83,84]. Seed-associated bacteria may be vertically transmitted and can influence early seedling development. In addition, disease-suppressive soils often harbor microbial communities enriched in taxa and metabolic pathways associated with pathogen inhibition [85,86].

A recent study examined drought-adapted rhizosphere environments as targeted sources for isolating stress-tolerant PGPR. Bacterial strains were collected from the rhizosphere of a cactus species growing under semi-arid conditions, and 246 isolates were obtained. All isolates were screened for plant growth-promoting traits, including IAA production, phosphate solubilization, ammonia production, and enzyme activities, followed by assessment of drought tolerance. Selected candidates were then tested on pepper plants under water stress conditions. Only a small proportion of isolates combined drought resistance with multiple beneficial traits, and several *Bacillus* strains demonstrated improved plant growth under limited moisture [87]. 

In addition to rhizosphere-derived isolates, seed-associated microbiomes represent another important natural source of beneficial microorganisms. Pal et al. (2022) [88] investigated maize seeds as reservoirs of beneficial endophytic bacteria and evaluated their role in early seedling development and pathogen protection. Twenty-three bacterial strains were isolated from maize seeds and identified by 16S 16S rRNA gene sequencing. Functional profiling assessed auxin production, phosphate solubilization, nitrogen fixation, and antifungal activity using dual-plate assays. Seed treatment with antibiotics reduced seedling growth, whereas reinoculation with selected isolates restored normal development, demonstrating a functional link between seed microbiota and plant performance. Several isolates inhibited fungal pathogens in vitro, and seed inoculation with *Bacillus velezensis* provided protection against *Fusarium verticillioides* [88]. The study offers strong experimental evidence that seed-associated microbiomes contribute to plant growth and defense and highlights a robust validation framework combining microbiome depletion and targeted reinoculation.

Building further on distinct ecological niches, the phyllosphere represents another important source of functional biocontrol strains. Shao et al. (2023) investigated bacterial isolates from tomato leaf surfaces for control of major foliar diseases caused by *Pseudomonas syringae* pv. tomato and *Alternaria solani*. Seven isolates were screened against multiple tomato pathogens, and two effective strains were identified as *Rhizobium* sp. and *Bacillus subtilis*. Both strains reduced disease development in detached-leaf assays and plant trials, and one strain activated the salicylic acid–dependent defense pathway. However, disease suppression varied across tomato cultivars, indicating a strong genotype effect [89].

Endophytes from desert halophytes have been investigated as sources of strains tolerant to saline–alkaline stress. Culturable bacteria isolated from roots of salt-adapted plants were screened for survival under high NaCl concentrations and alkaline pH, as well as for plant growth-promoting traits such as IAA production and phosphate solubilization. Several strains tolerated extreme conditions and improved wheat germination and seedling growth under salt stress [90]. In parallel, comparative analyses of wheat rhizospheres from disease-suppressive and conducive soils have examined whether specific environments uniquely enrich biocontrol traits. Community profiling and whole-genome sequencing of representative isolates revealed differences in microbial structure, but many plant growth-promoting and biocontrol genes were broadly distributed across soils. Although certain antibiotic biosynthesis genes were enriched in suppressive soils, phenotypic assays showed variable expression of predicted traits [91]. These findings indicate that source environment alone does not guarantee functional performance, emphasizing the need to combine community analysis, genomic screening, and phenotypic validation in strain selection.

### 3.2. Criteria for Strain Selection

Strain selection is a multi-criteria decision process. A microorganism that performs well in laboratory assays may not succeed in field conditions if it cannot persist in soil, compete with native microbiota, or effectively interact with a specific plant genotype [92,93,94]. Modern selection frameworks consider several interconnected factors [95,96]. These include functional potential, such as nutrient mobilization [97], phytohormone modulation, antagonistic activity, and induction of plant defense responses [98,99,100]. Ecological performance is also important, particularly colonization ability, persistence, and compatibility with specific crops or plant compartments. In addition, strains must demonstrate environmental robustness, including tolerance to relevant stresses [101,102]. Practical aspects such as growth characteristics and stability during production are essential for manufacturability, and biosafety and regulatory compliance remain critical components of final strain approval [103,104].

Building on multi-criteria strain selection strategies, Sidorenko et al. (2021) highlighted the importance of local ecological adaptation and functional complementarity in microbial selection. Bacterial isolates from long-term intensively managed soils were screened for nitrogen fixation and phosphate mobilization. Selected strains were tested individually and in binary combinations on wheat and barley seeds. The study showed improved germination energy and early seedling growth, with binary combinations outperforming single strains. The most effective treatments involved locally adapted and functionally complementary isolates [105].

Extending data-driven strain selection approaches, Voronina et al. (2024) evaluated whether laboratory PGP traits could predict wheat growth responses and soil changes. Soil isolates were screened for nitrogen fixation, phosphate solubilization, siderophore production, IAA synthesis, and antifungal activity, then identified by 16S rRNA sequencing. Multivariate analysis grouped strains according to dominant functional profiles, and all candidates were subsequently tested in wheat pot experiments [106].

Tsalgatidou et al. (2023) evaluated two endophytic *Bacillus halotolerans* strains for their compatibility and effectiveness as a combined biofertilizer–biocontrol consortium. The strains were applied individually and together through seed bio-priming and soil drenching, and their mutual compatibility, rhizosphere persistence, and impact on plant growth and gray mold suppression were assessed. The consortium significantly reduced disease severity compared with controls and was associated with activation of plant defense responses [107].

Beyond functional screening alone, compatibility with crop genotype and colonization persistence have emerged as critical selection filters. A recent study comparing resistant and susceptible soybean varieties used full-length 16S rRNA profiling to identify bacterial taxa enriched in resistant seeds. Isolates identified as *Bacillus altitudinis* were tested for antifungal activity and inoculated across different soybean genotypes. Disease resistance was restored only in compatible varieties that supported sustained colonization, with persistence in shoots lasting markedly longer in resistant lines than in susceptible ones [108]. The large-scale antagonism screening combined with genome-based analysis represents another modern selection pathway. Thousands of soil-derived isolates were screened for inhibition of *Fusarium pseudograminearum*, leading to identification of a highly antagonistic *Pseudomonas aeruginosa* strain. Genomic analysis revealed genes linked to siderophore and pyocyanin production, supporting mechanistic plausibility of disease suppression. While this approach illustrates the power of high-throughput screening coupled with whole-genome sequencing, it also highlights the importance of integrating biosafety and regulatory evaluation into strain selection, as species identity can influence deployment feasibility [109].

### 3.3. Screening and Validation Approaches

Screening and validation of agricultural microbial strains represent a stepwise and integrated process aimed at reducing uncertainty while maintaining biological relevance and cost efficiency [110,111]. The pipeline typically starts with niche-specific isolation aligned to the target stress or disease, followed by rapid in vitro screening for plant growth–promoting traits and antagonistic activity [112,113]. Selected strains are then tested for stress tolerance, survivability, and stability, and increasingly supported by genome-based analyses to identify functional genes and biosynthetic clusters [111,114]. Validation proceeds through plant-based assays and, in stronger studies, field trials and stability assessments [115]. Robust frameworks quantify effect size, connect phenotypes to mechanistic markers, and evaluate measurable agronomic outcomes [116,117].

In one representative example, Li et al. (2024) implemented a stress-aligned isolation strategy targeting maize grown in saline–alkaline soils. Candidate endophytic bacteria were enriched on medium containing high concentrations of NaHCO_3_, ensuring that the isolation filter reflected the intended field constraint. After 16S identification and whole-genome sequencing, physiological validation was performed under both normal and saline–alkaline stress conditions. The isolate demonstrated growth tolerance to high NaHCO_3_, enhanced seed germination under stress, reduced reactive oxygen species compared to stressed controls, increased chlorophyll content, improved root architecture, and confirmed root colonization [118].

A complementary strategy was demonstrated by Zhang et al. (2025), who combined phenotype-driven prioritization with comparative genomics to identify drought-relevant rhizosphere strains from winter wheat. Initial screening focused on classical plant growth–promoting traits such as phosphorus solubilization, nitrogen fixation, indole-3-acetic acid production, ACC deaminase activity, and siderophore synthesis. Pot experiments under normal and drought conditions revealed differential strain performance. Genome sequencing of selected candidates enabled comparative analysis of functional clusters associated with osmotic regulation and trehalose biosynthesis. One strain exhibiting both *treXYZ* and *ostAB* clusters uniquely enhanced root elongation under drought stress, supporting a mechanistic link between genomic potential and phenotypic performance [119].

Additional validation frameworks have emphasized quantitative disease metrics alongside genomic inference. In one study, an endophytic strain with antagonistic potential was evaluated through in vitro fungal inhibition assays, multi-trait PGP screening, stress tolerance testing across pH, temperature, and salinity gradients, and whole-genome mining of secondary metabolite clusters. Pot experiments quantified reductions in disease index relative to infected controls and chemical fungicide benchmarks. The integration of antagonistic efficacy, genomic biosynthetic predictions, and measurable reductions in pathogen burden provided a multi-layered validation model linking genotype, biochemical potential, and agronomic outcome [120].

Another investigation focusing on perennial crop systems isolated bacterial candidates from the root surface of date palm and screened them against a major *Fusarium* pathogen. Following antagonism-based triage, greenhouse trials demonstrated protective effects in seedlings. Genome analysis revealed multiple biosynthetic gene clusters associated with antifungal metabolites and carbohydrate-active enzymes capable of degrading fungal cell walls, as well as genes linked to nutrient mobilization and phytohormone synthesis [121].

A further example extended screening to a multi-level validation cascade culminating in field trials. After broad-spectrum in vitro antagonism testing, cell-free fermentation supernatants were evaluated for stability under heat, ultraviolet exposure, and pH variation, thereby treating laboratory outputs as proto-product attributes. Greenhouse trials demonstrated significant biocontrol efficiency, and field experiments confirmed high efficacy under natural conditions. By incorporating stability testing and real-field validation, this framework directly addresses the frequently observed gap between laboratory screening and agricultural deployment [122]. Current screening strategies favor integrated pipelines that link ecological isolation, functional testing, genomic analysis, and progressive plant validation. Aligning strain selection with target stresses and quantifying clear physiological or disease outcomes improves translational reliability. The addition of stability tests and field trials moves validation closer to practical agricultural application.

## 4. Molecular, Genomic, and Functional Characterization of Selected Strains

### 4.1. Taxonomic Identification and Genome Analysis

Taxonomic identification is the first “gate” in the development pipeline, because downstream efficacy, biosafety assessment and IP protection all depend on knowing exactly what organism is being commercialized. Traditionally, bacteria were identified using 16S rRNA gene sequencing, while fungi were often identified using ITS regions and additional loci. However, single markers can lack resolution among closely related taxa and may not reflect the evolutionary history across the whole genome. Recognizing this, the international journal of systematic and evolutionary microbiology community proposed minimal standards for using genome data in prokaryotic taxonomy, including requirements for genome quality and recommendations to use genome-scale metrics alongside phenotypic data [123]. These standards align well with industrial needs: a high-quality genome becomes a permanent reference that can be re-used for comparative analyses, rapid re-identification and regulatory dossiers.

Genome-based species delineation relies heavily on average nucleotide identity (ANI) and digital DNA-DNA hybridization (dDDH). Konstantinidis and Tiedje (2005) showed that genome similarity metrics reveal discontinuities that correspond to microbial species boundaries, supporting the use of whole-genome comparisons as a quantitative basis for defining species [124]. Building on this, Richter and Rosselló-Móra (2009) argued that ANI could act as a genomic “gold standard”, with ~95–96% ANI often approximating the traditional 70% DDH boundary [125]. In practice, developers of microbial inoculants increasingly compute ANI against reference genomes and type strains to confirm identity, avoid mislabeling and detect accidental strain substitution.

Beyond species boundaries, modern taxonomy is increasingly shaped by curated genome databases that standardize ranks from domain to species. The Genome Taxonomy Database (GTDB) proposed a genome-based taxonomy for Bacteria and Archaea that attempts to resolve inconsistencies in classical taxonomy by using standardized phylogenomic methods and relative evolutionary divergence [126]. For applied work, GTDB’s practical value is that it provides consistent placements even for taxa with confusing historical names, and it reduces the risk that a product’s taxonomy becomes outdated as nomenclature evolves. Tools such as GTDB-Tk enable rapid classification of new genomes against this framework and have been updated to be more memory-efficient while maintaining accuracy [127]. These tools are increasingly used in inoculant development workflows, particularly when companies sequence multiple candidate strains and need a standardized way to compare them.

**Figure 3:**
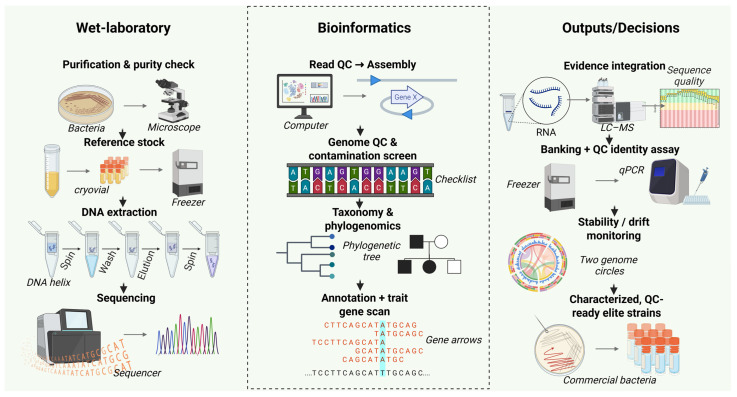
Workflow for genome-based selection and validation of elite microbial strains. The process includes strain purification and DNA extraction in the wet laboratory, genome sequencing and bioinformatic analysis (assembly, taxonomic identification, and functional gene annotation), followed by validation, quality control, and stability monitoring. The selected strains are then prepared for application and potential commercialization. Created with BioRender.com (License No. XS29DR9E4M).

In addition to taxonomy, strain identity for applied products must be “operationalized” into assays usable for QC. A common strategy is to anchor taxonomy using WGS, then design strain-specific markers for PCR/qPCR assays. Even when a strain’s species is correctly identified, regulatory and commercial practice often requires strain-level traceability because efficacy and safety can be strain-specific. In this context, WGS supports both deep phylogenetic classification and the practical design of identity tests that can be performed routinely on production batches (Figure 3)**.** The phylogenetic tree shown in the middle panel of Figure 3 represents the taxonomic verification step used to confirm strain identity prior to functional annotation and downstream characterization. The combination of genome-based taxonomy standards [123], quantitative similarity metrics [124,125], and global genome taxonomies [126,127] has therefore become a foundation for modern microbial product development.

### 4.2. Omics-Based Functional Insights

Once a strain is taxonomically anchored, genome analysis becomes a powerful lens for predicting and prioritizing candidate functions relevant to agriculture. This is particularly important because many plant-beneficial phenotypes are conditional: they depend on environmental cues, plant exudates, nutrient availability and competition within the microbiome. Genome sequencing provides a comprehensive inventory of genes, but functional interpretation requires robust annotation and comparative frameworks. Rapid, standardized annotation pipelines such as Prokka (2014) have helped make bacterial genome annotation routine and reproducible at scale [128]. Annotated genomes then support both hypothesis-driven analysis and discovery-driven analysis (mining genomes for novel biosynthetic gene clusters).

A major contribution of genome mining to agricultural microbiology is the systematic identification of biosynthetic gene clusters (BGCs) that encode specialized metabolites. Many successful microbial biocontrol agents (*Bacillus* and *Pseudomonas*) produce lipopeptides, polyketides, nonribosomal peptides and ribosomally synthesized peptides that suppress pathogens and help colonize plant surfaces. antiSMASH is a widely used platform for predicting BGCs and comparing them across genomes, and the release of antiSMASH 6.0 expanded supported cluster types and improved detection and comparison features [129]. However, prediction alone is insufficient to interpret novelty and likely activity. The MIBiG repository was created to curate BGCs of known function as a reference set; the MIBiG 2.0 update expanded and standardized these curated records, enabling better benchmarking and comparative analysis [130]. For inoculant development, a practical workflow is to mine candidate genomes with antiSMASH, map predicted BGCs to MIBiG-annotated clusters and then prioritize strains that encode BGCs associated with validated antifungal or antibacterial compounds while avoiding clusters linked to undesirable or regulated metabolites.

Functional gene analysis also supports claims related to plant nutrition and stress tolerance. For example, phosphate solubilization is commonly mediated by organic acid production and phosphatases, Rodríguez and Fraga (1999) reviewed phosphate-solubilizing bacteria and highlighted the role of organic acids and phosphatases in mobilizing mineral and organic phosphorus, respectively [131]. Ethylene modulation through ACC deaminase is another well-established trait that can improve plant tolerance to abiotic stress. Orozco-Mosqueda et al. (2020) [132] reviewed the role of bacterial ACC deaminase in promoting plant growth under salt stress and emphasized its relevance across rhizospheric and endophytic bacteria. Such reviews, combined with comparative genomics, allow developers to check whether candidate strains carry canonical genes (*acdS*) and associated regulatory elements, which helps explain performance differences among strains that may appear similar in simple in vitro screens [132,133,134,135].

A critical limitation of “gene presence” predictions is that expression and metabolite production depend on growth conditions. This is where omics approaches provide essential validation. Multi-omics reviews have emphasized that transcriptomics and proteomics can reveal how plant-beneficial microbes switch between soil survival, rhizosphere colonization and antagonism, while metabolomics can confirm the actual production of hormones, siderophores and antimicrobial compounds in planta [136]. More broadly, omics data help identify biomarkers that correlate with field efficacy, such as the expression of colonization genes, stress response pathways or production of specific lipopeptides during plant interaction. Wang et al. (2023) reviewed how synthetic microbial communities, and functional pathways can be linked to biological control outcomes, illustrating how mechanistic insights from omics can inform rational strain selection and consortium design [137].

In sum, genome analysis serves both as a “catalogue” of potential functions and as a platform for discovery. Practical inoculant development increasingly integrates standardized annotation [128], BGC mining and curation [129,130], targeted trait gene analysis for nutrient and stress pathways [131,132], and omics validation under plant-relevant conditions [136,137].

### 4.3. Genetic Stability and Trait Preservation

Even when a strain is correctly identified and its genome suggests valuable functions, commercialization introduces a less visible risk: genetic and phenotypic drift. Repeated subculturing, long fermentation runs, selection pressures in bioreactors, and stress during formulation can lead to mutations, plasmid loss or shifts in gene regulation that affect efficacy. For agricultural products, drift can manifest as reduced colonization, weaker antagonism, altered metabolite profiles or changes in stress tolerance often detected only when field performance declines.

From a microbiological perspective, drift is influenced by the strain’s genome architecture and by the production process. *Bacillus* biocontrol strains, for instance, often rely on large gene clusters for lipopeptide synthesis; mutations in regulatory networks can reduce metabolite production without strongly affecting growth. Similarly, *Pseudomonas* strains may lose plasmids carrying key traits if they impose a metabolic burden in rich media. Genome sequencing enables direct surveillance: by comparing WGS data from master cell banks, working cell banks and production batches, developers can detect SNP accumulation, structural variation or loss of genomic regions. The availability of global genome-based taxonomies and standardized genomic methods [123,126] strengthens these comparisons because genomes are evaluated against consistent quality and contamination criteria.

Microbial preservation strategies are also tightly linked to genetic stability. Prakash et al. (2013) reviewed microbial preservation practices and emphasized that preservation is not only about survival but also about maintaining functional characteristics over time; they highlighted that inappropriate storage or repeated passaging can alter phenotypes and recommended controlled banking strategies [133]. In industrial practice, a two-tier banking system combined with defined limits on passage number is commonly used. Genomic confirmation can be performed at critical points: after banking, after scale-up validation, and periodically during routine production.

Stability considerations also connect to the broader challenge of translating a laboratory biocontrol strain into a “bankable” commercial product. Teixidó et al. (2022) discussed production, formulation, packaging and shelf life as integrated aspects of successful biocontrol agent development, noting that conditions during growth and formulation can influence long-term stability and efficacy [134]. Their review underscores that stability is not purely genetic: physiological state, stress history and formulation matrix can all affect performance. This motivates combined genetic–physiological monitoring, where genomics verifies identity and stability while physiological assays quantify traits such as spore percentage, metabolite production, or stress tolerance.

Finally, genetic stability assessment benefits from connecting genomic changes to functional outputs using omics. Multi-omics studies highlight that shifts in gene expression and metabolite production can occur even without large genetic changes, for example due to epigenetic regulation or persistent physiological states. Therefore, stability programs increasingly include not just genotyping but also functional profiling under standardized conditions relevant to product application. Reviews on multi-omics in plant growth-promoting microorganisms emphasize that integrating genomics with transcriptomics/proteomics/metabolomics can reveal “early warning” indicators of performance loss [136]. Overall, published work supports a best-practice framework: establish a high-quality reference genome, maintain controlled banking with minimal passages, monitor genetic integrity with periodic WGS comparisons, and verify that key plant-beneficial functions are preserved through targeted assays and, when feasible, omics-based profiling [133,134].

## 5. Optimization of Cultivation and Fermentation Processes

### 5.1. Culture Media and Growth Parameters

**Figure 4:**
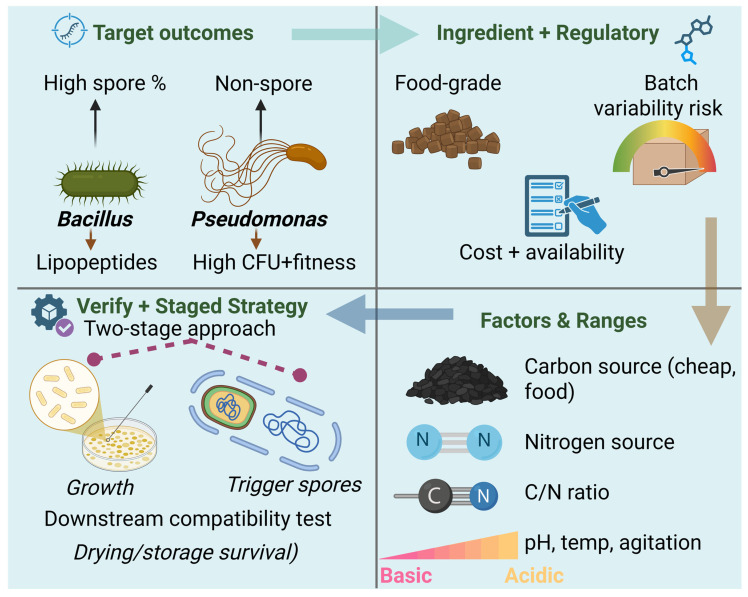
Strategy for cultivation and fermentation optimization of microbial inoculants. Target outcomes are aligned with ingredient and regulatory constraints, including food-grade substrates, cost, availability, and batch variability risk. Key process factors carbon and nitrogen sources, C/N ratio, pH, temperature, and agitation are optimized using a staged approach that separates biomass growth from trait induction. Downstream compatibility, including drying and storage survival, is incorporated to ensure formulation-ready performance. Created with BioRender.com (License No. MJ29DULUBI).

Media optimization typically begins with selecting inexpensive, food-grade carbon and nitrogen sources that support high biomass or propagule yield without repressing key traits [9,138]. For *Bacillus* biocontrol strains, the target is often a high spore percentage combined with sustained production of lipopeptide antimicrobials; for *Pseudomonas* or other non-spore formers, the goal is high cell density with robust physiological fitness (Figure 4). A consistent theme across studies is that single-factor optimization is inefficient and can miss interactions between nutrients. Design-of-experiments (DoE) methods provide an efficient alternative by screening many factors and quantifying interactions in a limited number of experiments. Mandenius and Brundin (2008) reviewed DoE applications in bioprocess development and argued that factorial designs and response surface methodologies can accelerate optimization while improving process understanding [139]. Their review is particularly relevant for agricultural inoculants because it highlights how DoE can be applied not only to media composition but also to unit operations and downstream handling.

Recent studies have applied these concepts directly to microbial biocontrol production. Shi et al. (2024) used Plackett–Burman and Box–Behnken designs to optimize medium components and culture parameters for *Bacillus velezensis* BHZ-29, improving viable counts and antibacterial potency simultaneously [140]. Similarly, Liu et al. (2025) applied response surface methodology to optimize fermentation conditions for *Bacillus amyloliquefaciens*, demonstrating how statistical optimization can identify balanced conditions that support both growth and bioactivity [141]. Such case studies show that optimization must consider multiple outputs: maximizing CFU alone may not maximize antagonistic activity if metabolite synthesis requires specific nutrient limitations or stress cues.

Optimization also often includes deliberate manipulation of C/N ratio, micronutrients and pH control to steer physiology. For example, sporulation in *Bacillus* can be promoted by nutrient limitation and controlled pH, while secondary metabolite production can be linked to specific amino acid precursors. Vahidinasab et al. (2022) characterized a *Bacillus velezensis* strain producing multiple lipopeptide families and reported that amino acid supplementation modulated surfactin, iturin A and fengycin titers, illustrating how targeted nutrient strategies can influence product potency [142]. These findings echo broader industrial fermentation principles: the same medium that maximizes biomass may not maximize the desired functional “payload”. Therefore, optimization frequently uses staged strategies first building biomass, then triggering sporulation or metabolite synthesis.

For non-spore-forming rhizobacteria, media formulation must also consider downstream formulation compatibility. Bisutti and Stephan (2020) demonstrated that fermentation temperature and duration affected survival of a bacterial biocontrol agent during drying and storage, emphasizing that “upstream” choices can strongly influence “downstream” viability [143]. Overall, published work supports an integrated optimization mindset: nutrient composition and culture parameters should be tuned not only for growth but also for physiological robustness and the intended downstream processing route [139,143].

Beyond maximizing yields, media optimization in agricultural biotechnology must consider ingredient availability, price volatility and regulatory acceptability. Many papers report successful replacement of laboratory-grade components with agro-industrial by-products, which can lower costs but also introduce batch variability. DoE-based screening is well suited to these situations because it can evaluate robustness to ingredient variation and identify “forgiving” formulations that maintain performance across expected raw-material ranges (Table 2) [139].

### 5.2. Scale-Up from Laboratory to Industrial Production

Fermentation technology choices reflect the organism and the intended product form. Submerged fermentation (SmF) in stirred-tank reactors is common for bacteria because it offers tight control of pH, dissolved oxygen (DO) and temperature, and it supports aseptic operation at scale. Solid-state fermentation (SSF) is widely used for fungi (e.g., *Trichoderma*, *Beauveria*) because it can generate high conidial yields on low-cost substrates and can mimic natural growth conditions. A comprehensive review by Sala et al. (2019) summarized advances in SSF for fungal biocontrol agents using organic solid waste, highlighting how substrate selection, moisture control and aeration shape spore yields and product quality [144]. More recent SSF work has explored alternative substrates and process configurations: Bulgari et al. (2023) reported SSF of *Trichoderma* on agricultural digestate mixtures to produce bioactive molecules suitable for biostimulant formulations, illustrating the potential of circular-economy substrates [145]. Narwade et al. (2023) developed a simple earthen-vessel SSF approach for *Trichoderma* spore production, emphasizing robustness and cost effectiveness for resource-limited settings [146]. Collectively, these studies show that SSF optimization must account for heterogeneous conditions and for downstream drying requirements.

For bacterial SmF, scale-up hinges on oxygen transfer, mixing and heat removal. Garcia-Ochoa and Gómez reviewed oxygen transfer and uptake in stirred-tank bioreactors and described how the volumetric oxygen transfer coefficient (kLa) depends on agitation, aeration and broth rheology parameters that change during growth as cells produce exopolymers or form aggregates [147]. This matters for inoculants because many beneficial bacteria form biofilms or produce surfactants that alter mass transfer, while filamentous fungi create complex morphologies that affect viscosity. Scale-up strategies often rely on maintaining a key engineering parameter to preserve physiological conditions. However, published case studies indicate that a single parameter rarely captures the full complexity; instead, scale-up is frequently iterative, using pilot-scale runs and in-process monitoring.

Industrial production of *Bacillus*-based products often targets high spore yield and, in some cases, co-production of lipopeptide antimicrobials. Biermann et al. (2023) described bioprocess development for endospore production with high sporulation efficiency in bioreactors, showing that pH control and parameter robustness testing can markedly improve sporulation performance [148]. Soliman et al. (2022) reported stirred-tank production of a *Bacillus velezensis* strain for biocontrol purposes, illustrating practical challenges such as balancing growth and product formation in batch fermentation [149]. In parallel, Tang et al. (2023) demonstrated that biofilm-based fermentation could increase iturin A productivity compared with suspended cultures, suggesting that engineering strategies exploiting biofilm physiology may enhance metabolite yields for certain *Bacillus* strains [150].

Taken together, the literature indicates that fermentation and scale-up decisions should be made jointly with the desired product form and mechanism of action. SSF can be advantageous for fungal spore products and for certain bacterial spore processes, while SmF provides control and consistency for bacterial inoculants and metabolite-driven products. Across both platforms, rigorous monitoring of physiological and engineering variables is essential to reduce the risk of scale-up failure and to ensure batch-to-batch consistency [147,148].

Scale-up studies also highlight the importance of selecting an appropriate cultivation mode. Fed-batch strategies, widely used in industrial biotechnology, can help maintain substrates at non-inhibitory levels, avoid overflow metabolism and extend production phases. While the reviewed case studies largely focus on batch fermentations, several authors discuss fed-batch as a logical next step for increasing titers once basic growth and control strategies are established [142]. In practice, implementing fed-batch requires reliable online measurements and control policies that link feeding to DO, pH, off-gas analysis, or biomass estimates [148].

Another recurring theme is that scale-up should preserve not only growth rate but also product-relevant physiological “signatures”, such as a characteristic DO drop linked to secondary metabolism onset or a pH profile associated with sporulation. The oxygen transfer review by García-Ochoa and Gómez (2009) emphasizes that broth properties can change over time and alter effective kLa, so maintaining a target kLa may require dynamic adjustment of agitation and aeration rather than fixed setpoints [147]. Case studies of *Bacillus* biocontrol production in stirred tanks report common practical solutions: antifoam management, staged aeration, and using DO-stat control to keep cultures within a desired physiological window [149].

### 5.3. Stress Resistance During Mass Cultivation

The final commercial value of a microbial product depends strongly on its ability to survive downstream processing and to remain viable during storage and transport. Stress resistance is therefore a targeted trait during process development. For spore-forming bacteria, promoting sporulation can dramatically increase stability because spores resist heat, desiccation and UV. Zhao et al. (2019) used a two-stage solid-state fermentation strategy to enhance *Bacillus subtilis* growth and sporulation, showing that staged nutrient and process control can steer development of resilient propagules [151]. For *Bacillus* produced in submerged fermentation, optimizing pH control and nutrient limitation can increase spore percentages and reduce the need for harsh drying conditions [148].

For non-spore-formers, stress resistance is often improved by controlling physiological state at harvest and by adding protectants during drying. The work of Bisutti and Stephan (2020) illustrates this connection clearly: changing fermentation temperature and harvest time affected the shelf life and viability of freeze-dried *Pseudomonas fluorescens* Pf153 and, in bioassays, influenced biocontrol efficacy on leaves [143]. These findings support a broader principle in fermentation science: cell physiology at the time of harvest can determine how well a culture withstands dehydration and oxidative stress during storage.

Downstream stabilization is frequently strengthened by compatible solutes and protein protectants. Krzymińska and Kowalska (2024) evaluated trehalose and monosodium glutamate as additives during freeze-drying and reported improved viability and maintenance of antagonistic properties of yeast biocontrol isolates after storage and rehydration [152]. Although the study focused on yeasts, similar mechanisms—membrane protection, reduced protein denaturation, and improved rehydration—are relevant for many agricultural inoculant organisms. For metabolite-driven *Bacillus* products, process design can also exploit microbial lifestyle strategies such as biofilm-based fermentation systems.

A key message from the literature is that stress resistance should be treated as an optimization objective, not as an afterthought. Upstream parameters, midstream choices, and downstream steps interact to determine final viability. Studies that integrate these steps optimizing culture conditions specifically for downstream survivability—tend to report more stable CFU counts over storage than studies that optimize biomass alone [139,143]. For product developers, this implies that viability after drying, and storage should be included as a key response variable in DoE and scale-up experiments from the first pilot runs [139].

**Table 2:** Key cultivation and fermentation optimization levers for microbial inoculant production and reported effects.

Optimization	Approach	Reported Effect	Ref.
Design-of-experiments (DoE) for upstream tuning	Screening + response-surface designs	Identifies dominant factors and interactions; supports simultaneous optimization of biomass, sporulation, or metabolite titers	[139,140,141]
Carbon/N sources and C:N ratio	Molasses/glucose/glycerol with yeast extract/peptone or defined salts; adjust C:N for growth vs. secondary metabolism	Strong driver of growth rate and lipopeptide/antimicrobial production in *Bacillus*-type inoculants	[140,141,142]
pH and temperature profiles	Maintain strain-specific optima; use controlled pH or staged temperature to steer sporulation	Affects enzyme activity, sporulation, and later stress tolerance; temperature/time during fermentation can influence post-drying survival	[143,148,151]
Aeration, agitation, and dissolved oxygen (DO)	Set agitation/aeration to achieve target OTR/kLa; DO control at scale	Oxygen transfer is a frequent scale-up bottleneck; insufficient OTR reduces yields and may shift product quality (e.g., spore %)	[147,149]
Scale-up criteria and reactor strategy	Scale by kLa, power input/volume, or mixing time; validate in pilot stirred-tank runs	Maintains comparability across scales while managing shear and gradients; improves reproducibility of industrial batches	[147,148,149]
Sporulation/propagule formation triggers	Nutrient limitation, two-stage cultivation, or SSF phases to induce spores	Higher spore fractions often increase storage stability and field robustness compared with purely vegetative biomass	[138,151]
Solid-state fermentation (SSF) platform	Choice of substrate, moisture/temperature control, and mixing/bed design	SSF can improve spore yields and reduce costs/water use, but requires careful control of heterogeneity and contamination risk	[144,145,146]
Biofilm/immobilized-cell fermentation	Two-compartment/biofilm reactors or immobilization supports	Biofilm physiology can enhance lipopeptide titers and sometimes stabilizes productivity under process stresses	[150]
Harvest timing and downstream survivability targets	Select harvest phase; include protectants for drying; validate CFU after drying/storage	Treats viability after drying as a response variable; improves final CFU stability and retained antagonistic activity	[139,144,152]

## 6. Formulation, Quality Control, and Biosafety of Biological Products

### 6.1. Formulation and Stabilization Strategies

Carrier materials are the physical matrices that deliver microorganisms to the field and help protect them during storage and application. Classic carrier-based inoculants used peat, lignite, talc, vermiculite or compost-like materials; newer formulations include liquid suspensions, polymeric microbeads, and biochar-based carriers. The selection of a carrier is multi-criteria: it must maintain viability during storage, be compatible with the intended application method, allow predictable release of cells or propagules, be safe and acceptable under regulations, and be economical and available at scale [153,154].

Malusá et al. (2012) reviewed technologies used to prepare inocula of beneficial microorganisms and emphasized that carrier choice is closely linked to the microorganism’s physiology and to the target crop and application pathway [153]. For example, spore-forming *Bacillus* products can tolerate a wider range of low-moisture solid carriers than non-spore-forming Pseudomonas, which often benefits from protective liquids or encapsulation. Khan et al. (2023) further highlighted that bioformulations should be viewed as engineered systems where carriers, additives and microbes are co-designed to improve colonization and plant response, rather than as passive mixtures [155].

A rapidly growing research direction is the evaluation of alternative, sustainable carriers that reduce reliance on peat and enable circular-economy supply chains. Shabir et al. (2026) reviewed alternative carrier materials for plant growth-promoting rhizobacteria (PGPR) and discussed how carriers such as composts, biochar, and agricultural residues can influence microbial survival and functional expression by altering pH, nutrient availability, and water retention [156]. Empirical studies echo this: Sohaib et al. (2020) compared carrier-based, multi-strain biofertilizer formulations and showed that carrier properties can interact with microbial consortia to influence plant responses under stress [157].

Although many carriers can maintain high viable counts shortly after preparation, the critical differentiator is often longer-term stability and release behavior. Reviews emphasize practical carrier attributes that correlate with stability: low water activity (to slow metabolic exhaustion), moderate buffering capacity, low levels of antimicrobial contaminants, and a porous structure that supports oxygen diffusion without excessive drying [154,156]. Therefore, modern carrier selection increasingly includes accelerated aging tests and compatibility tests with seed treatments and adjuvants, in addition to simple initial CFU counts.

### 6.2. Carrier Materials, Additives, and Shelf Life

Stabilization methods aim to preserve viability and functionality over months rather than days. Approaches controlling physiological state at harvest, drying to a stable water activity using freeze-drying or spray-drying, adding protectants such as sugars and proteins, and physical encapsulation that creates a microenvironment around cells. A central insight from formulation research is that stabilization is rarely universal: the optimal strategy depends on the microorganism, the intended carrier and the application route [158].

Encapsulation in alginate has a long history in inoculant technology and remains a benchmark method for sustained release and protection. Bashan (1986) developed uniform alginate beads as synthetic inoculant carriers and showed that bead hardening treatments could control bead strength, bacterial release rate and survival time in soil, enabling slow and stable delivery of plant-beneficial bacteria [159]. Alginate encapsulation can also facilitate co-formulation with protectants. Bashan and Gonzalez (1999) reported long-term survival of encapsulated *Azospirillum* in dry alginate beads, demonstrating that, with appropriate drying and storage, encapsulation can support extended shelf life beyond what is typical for simple carrier powders [160].

Later work refined alginate approaches by enriching the bead microenvironment with organic substances. Young et al. (2006) encapsulated a *Bacillus subtilis* plant growth-promoting isolate in alginate beads enriched with humic acid and observed high viability with minimal loss over several months, alongside steady release of cells across pH conditions relevant to soils [161]. Reviews of encapsulation and microencapsulation for agricultural microorganisms emphasize similar strategies adding humic substances, nutrients, or protective polymers to improve storage stability and release [6]. More recent studies also extend encapsulation to consortia and to targeted bioactive traits. For example, Mushtaq et al. (2025) developed alginate beads containing auxin-producing bacteria and reported improved plant growth responses compared with free-cell applications under their experimental conditions [162].

Drying and packaging are complementary to encapsulation. Freeze-drying typically yields high survival for many bacteria and yeasts but requires protectants to reduce ice and dehydration damage, while spray-drying is cheaper and continuous but can impose higher thermal stress. The packaging study by Costa et al. (2002) on freeze-dried Pantoea agglomerans demonstrated that package type and storage conditions substantially influenced viability and biocontrol efficacy, illustrating that packaging is part of the formulation system, not merely a logistics detail [163]. For agricultural products intended for warm supply chains, this has practical implications: moisture-barrier packaging and desiccants can be as important as the choice of drying method.

### 6.3. Quality Control and Standardization

Quality control (QC) provides evidence that a microbial product matches its specification: correct strain identity, viable count at release, purity, and predicted shelf life. Herrmann and Lesueur (2013) argued that quality variability is a major reason for inconsistent farmer outcomes and emphasized the need for better QC systems worldwide, including standardized microbial counts and contamination thresholds [154].

Viable count is traditionally measured by plating and CFU enumeration. This is simple and regulatory-friendly, but it can underestimate viable but non-culturable cells and can be slow for spores or slow-growing organisms. As a complement, molecular and cytometric methods have been increasingly explored. Viability PCR approaches using membrane-impermeable dyes combined with qPCR can preferentially quantify intact cells and have been reviewed as practical tools for microbiological quality assessment when plating is limiting [163,164]. For inoculants, the key advantage is speed and potential strain specificity: qPCR primers can target strain-unique markers identified from genome data, enabling simultaneous identity confirmation and approximate viable-load quantification in complex carriers. However, the literature cautions that viability dyes can behave differently across matrices (powders, biochar, alginate) and species, so method validation against plating remains necessary [164].

Flow cytometry offers another route to rapid enumeration and physiological profiling. Although not yet universal in agricultural inoculant QC, microbial flow cytometry is widely used in water and food microbiology and is increasingly proposed for bioproduct monitoring because it can quantify total cells, membrane integrity and metabolic activity within minutes. Where flow cytometry is used, a practical approach is to define acceptance criteria based on a combination of total counts and a viability gate, then correlate these metrics with plating during method development. Such dual approaches can strengthen shelf life studies because they reveal whether losses are due to cell death, aggregation, or loss of culturability.

QC also includes identity assurance beyond counts. For products registered as specific strains, identity can be supported by sequencing-based fingerprints and by strain-specific PCR assays. This is particularly important for consortia, where contamination or drift can change the relative abundance of members. Reviews of bioformulations emphasize that consortium products require additional QC layers: member verification, ratio stability and antagonism testing to ensure compatibility over storage [155,156].

Shelf life testing should be treated as a controlled experiment rather than an afterthought. Accelerated stability studies at elevated temperature and humidity can rapidly compare formulations, but they must be linked to real-time studies under realistic storage conditions. The packaging work of Costa et al. (2002) illustrates that storage environment can dominate stability outcomes even for the same dried biomass, supporting the inclusion of packaging and moisture control in shelf life protocols [163].

### 6.4. Biosafety and Regulatory Considerations

For microbial biopesticides, many jurisdictions require more formal risk assessment like chemical plant protection products. In the United States, the Environmental Protection Agency (EPA) provides microbial pesticide test guidelines covering product analysis, toxicology, and environmental fate, which have become reference points for developers planning data packages. Even when products are marketed as biologicals, regulators generally expect evidence that the microorganism does not pose unacceptable risks to humans, livestock, beneficial insects or the environment. Reviews also emphasize that secondary metabolites can complicate risk assessment: a strain may be non-pathogenic, yet its metabolite profile may include compounds requiring evaluation depending on concentration and exposure [165]. This connects directly back to earlier sections: genome mining and metabolomics can help anticipate metabolite-related concerns, while stability monitoring helps ensure that a product remains consistent with the strain evaluated in the regulatory dossier. For biofertilizers and plant biostimulants, regulatory approaches differ across countries and are still evolving. Malusá and Vassilev (2014) discussed the need for coherent legal frameworks for biofertilizers and argued that quality standards, labeling rules, and traceability are essential for market development [166,167].

Finally, biosafety is not only about regulatory compliance but also about responsible deployment. Good manufacturing practice (GMP) controls clean facilities, validated sterilization, contamination monitoring reduce the chance of distributing unintended microbes. Clear instructions for use (application rate, timing, storage conditions) reduce misuse that could lead to poor performance or environmental release beyond intended conditions. Across the literature, the consistent recommendation is to integrate biosafety into development rather than treating it as a final hurdle: select strains with clear identity and safe-use precedents, design formulations that reduce contaminant growth, and build QC systems that document identity and purity over the product lifecycle [166,167].

## 7. Challenges and Knowledge Gaps

**Figure 5:**
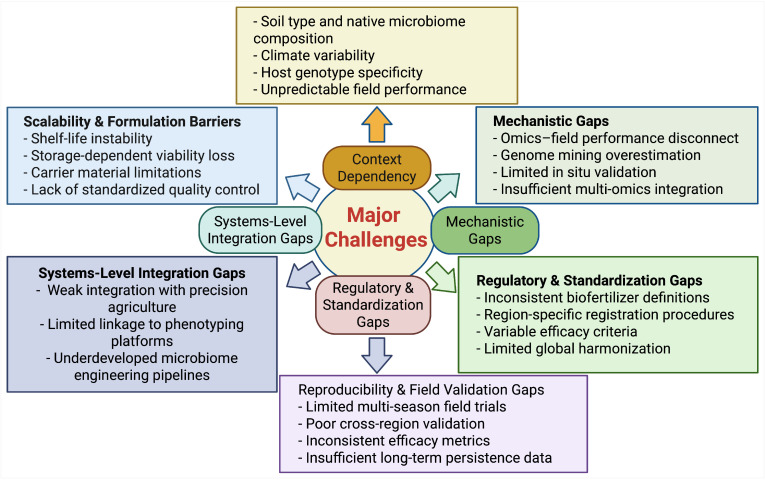
Conceptual overview of major challenges and knowledge gaps limiting the field-level performance and large-scale adoption of PGPMs and microbial biocontrol technologies. Created with BioRender.com (License No. ZE29E2GSIO).

Despite rapid progress in PGPMs and microbial biocontrol technologies, several critical challenges continue to limit consistent field-level performance and large-scale adoption [168]. First, context dependency and inconsistent field efficacy remain major bottlenecks. Increasing evidence shows that microbial inoculant performance is strongly influenced by soil type, native microbiome composition, climate, and host genotype. Recent studies emphasize that rhizosphere assembly dynamics and niche preference significantly shape microbial persistence and function [169]. Likewise, microbial community-level interactions determine whether introduced strains integrate, compete, or collapse within native consortia [170]. Although synthetic communities show promise in stabilizing functional outputs [171,172], predictive rules governing community compatibility and long-term resilience remain insufficiently understood (Figure 5). Despite promising experimental results, many microbial products may show inconsistent performance under real agricultural conditions due to variations in soil properties, climate, native microbiota, and crop genotype. Therefore, long-term multi-season validation and improved strategies for selecting strains adapted to specific agroecosystems are required.

Second, mechanistic resolution is still incomplete. While omics-driven approaches have expanded our understanding of functional traits and secondary metabolite biosynthesis [136,173], translating genomic potential into reproducible phenotypic outcomes under field conditions is still challenging. Genome mining and pathway prediction often overestimate in situ expression, and integrative multi-omics under realistic agronomic conditions remains scarce.

Third, scalability and formulation stability present persistent industrial barriers. Although advances in fermentation optimization and bioformulation strategies have improved cell viability and metabolite production, maintaining biological activity during storage and after soil application is still problematic [155,156]. Encapsulation technologies and alternative carrier materials offer promising solutions, yet standardization of shelf life testing and performance validation is lacking.

Fourth, regulatory harmonization and quality control gaps continue to slow commercialization. Recent analyses highlight inconsistencies in biofertilizer definitions, registration procedures, and efficacy requirements across regions [34,42]. The absence of globally aligned regulatory frameworks complicates market expansion and investor confidence.

Finally, systems-level integration remains underdeveloped. Linking microbiome engineering with precision agriculture, phenotyping platforms, and climate-adaptive management strategies is still in its infancy. Data-driven synthetic microbiology approaches [31,174] represent a promising frontier, yet standardized pipelines for translating computational design into agronomic validation are urgently needed.

Addressing these knowledge gaps requires interdisciplinary integration across microbiology, systems biology, agronomy, bioprocess engineering, and regulatory science to ensure that microbial technologies move from experimental promise to reliable, climate-resilient agricultural solutions.

## 8. Conclusions and Future Perspectives

Microorganism-based biological products for agriculture require an integrated, systems-level development framework rather than isolated trait optimization. Across biofertilizers, biopesticides, PGPMs, and microbial consortia, functional efficacy depends not only on measurable biochemical traits such as nutrient mobilization, secondary metabolite production, or phytohormone modulation but also on ecological compatibility, colonization persistence, genetic stability, and production scalability. Genome-based taxonomy, comparative genomics, and omics-driven functional analyses provide robust tools for improving strain selection and reducing early-stage uncertainty; however, laboratory validation alone remains insufficient to predict field performance. Reliable biomass production does not arise from strain selection alone. It depends heavily on fermentation optimization, staged cultivation regimes, and statistically informed process design to secure reproducible yields and stress-resistant propagules. Yet even technically optimized cultures may fail without robust formulation engineering and carrier systems, since downstream stability ultimately defines shelf life and agronomic consistency. Quality control systems based on strain-level identity verification, viability assessment, and genetic or phenotypic drift monitoring are increasingly recognized as central determinants of product reliability. Despite technological advances, variability in field performance remains a major constraint, underscoring the need for predictive indicators that connect molecular traits with environmental and host-specific contexts. Future efforts should prioritize integration of genomics, transcriptomics, metabolomics, and ecological modeling to establish predictive strain performance frameworks. Rational consortium design, improved colonization tracking tools, and harmonized regulatory standards will enhance translational robustness. Such rational consortium design should consider functional complementarity among strains, ecological compatibility, and niche differentiation within the rhizosphere. Genome-informed trait analysis, metabolic interaction modeling, and synthetic community experiments are increasingly used to identify synergistic microbial combinations and improve stability under field conditions. In addition, colonization and persistence of introduced strains can be monitored using strain-specific qPCR markers, metagenomic sequencing, fluorescent labeling, and reporter gene systems. Coupling fermentation engineering with formulation science and technology readiness assessment can further accelerate commercialization. Sustained progress in microorganism-based agricultural solutions will therefore depend on coordinated innovation across microbiology, bioinformatics, bioprocess engineering, agronomy, and regulatory science to ensure reproducibility, scalability, and long-term sustainability under diverse agroecosystem conditions.

## Figures and Tables

**Table 1:** Integrated research approaches in microbial biocontrol and biopesticide development, from strain validation to formulation engineering.

Organism/Product Type	Experimental Approach	Key Results	Ref.
*Bacillus velezensis* SDTB038 (bacterial biocontrol)	Whole-genome sequencing; in vitro inhibition; pot assays; stress/ROS and gene expression readouts	Genomic secondary metabolite clusters; disease suppression in planta; coupled growth/PGP traits	[51]
*B. amyloliquefaciens* E2 (screened via microbiome induction)	Pathogen-induced rhizosphere reconstruction; antagonist isolation; colonization quantification; pot efficacy; defense markers	High inhibition and colonization; high control efficacy and defense gene upregulation	[52]
*Beauveria bassiana* conidia microparticles (spray-dried)	Polymer ratio optimization; physicochemical QC; release; thermal stress; insect bioassay	High conidia viability and loading; improved thermal tolerance; higher insect mortality vs. non-formulated	[53]
*Beauveria bassiana* in CMC beads (ionic gelation)	Compare Al vs. Fe cross-link; SEM-EDX/FTIR/TGA/XRD; UV & heat protection metrics	Al-beads improved loading and strong UV/heat protection; microstructure-performance linkage	[54]
*Trichoderma* conidia in oil-in-water emulsion	Formulation screening; dual-culture inhibition; in vivo tissue tests; storage tests	Reduced disease severity in vivo; improved viability at cool storage; spray-compatibility focus	[55]
*Bacillus subtilis* + *B. amyloliquefaciens* mix (disease suppression + microbiome shift)	Field/pot-style agronomic + rhizosphere microbiome assessment (paper focus)	Suppressed *Rhizoctonia* disease; improved rhizosphere microbiome + potato growth/yield indicators	[56]
*Trichoderma atrobrunneum* conidia (biopolymer-based emulsion stabilization)	Biopolymer/nanoparticle stabilization; formulation screening; performance/viability metrics	Demonstrated stabilization strategy for conidia; addresses shelf life & formulation compatibility	[57]
*Bacillus velezensis* L1 VOCs (postharvest biocontrol route)	VOC profiling + in vitro/in vivo (postharvest) inhibition assays	VOCs inhibited multiple fungal diseases; supports metabolite/VOC-mediated biocontrol concept	[58]
Rhizosphere microbiome comparison (healthy vs. infected) → biocontrol potential	Community composition/functional comparison under disease pressure	Identifies “health-associated” microbiome signals; supports ecology-driven candidate selection	[59]
Rhizosphere microbiome analysis + isolation of biocontrol bacteria (sugarcane root rot severity gradient)	Microbiome profiling + isolation of candidate strains; disease suppression evaluation	Links disease severity with microbiome; isolates promising suppressive strains	[60]

## Data Availability

Data are available on request.

## Abbreviations

ACC	1-aminocyclopropane-1-carboxylate
AMF	Arbuscular Mycorrhizal Fungi
CFU	Colony Forming Unit
DoE	Design of Experiments
EPA	Environmental Protection Agency
IAA	Indole-3-Acetic Acid
ISR	Induced Systemic Resistance
NFC	Nitrogen-fixing cyanobacteria
OTR	Oxygen Transfer Rate
PGPM	Plant Growth-Promoting Microorganisms
PGPR	Plant Growth-Promoting Rhizobacteria
PSB	Phosphate-Solubilizing Bacteria
SSF	Solid-State Fermentation
VOC	Volatile Organic Compounds
